# ﻿New species and newly recorded species of *Anisandrus* Ferrari, 1867 ambrosia beetles from Thailand (Coleoptera, Curculionidae, Scolytinae, Xyleborini)

**DOI:** 10.3897/zookeys.1182.105449

**Published:** 2023-10-20

**Authors:** Wisut Sittichaya, Sarah M. Smith, Roger A. Beaver

**Affiliations:** 1 Agricultural Innovation and Management Division, Faculty of Natural Resources, Prince of Songkla University, Songkhla, 90110, Thailand Prince of Songkla University Songkhla Thailand; 2 Department of Entomology, Michigan State University, 288 Farm Lane, 243 Natural Science Bldg, East Lansing, MI 48824, USA Michigan State University East Lansing United States of America; 3 161/2 Mu 5, Soi Wat Pranon, T. Donkaew, A. Maerim, Chiangmai 50180, Thailand Unaffiliated Chiangmai Thailand

**Keywords:** Key, new records, Oriental region, Thai fauna

## Abstract

Five new species, *Anisandrusmontanus***sp. nov.**, *A.phithakpa***sp. nov.**, *A.tanaosi***sp. nov.**, *A.triton***sp. nov.**, and *A.uniseriatus***sp. nov.** are described from Thailand. *Anisandruscarinensis* (Eggers, 1923) is reported from Thailand for the first time and *A.apicalis* is removed from the Thai fauna. With the inclusion of the species described and recorded here, the diversity of *Anisandrus* is increased to 40 species, of which 11 occur in Thailand. A synoptic list and a key to the *Anisandrus* of Thailand are presented.

## ﻿Introduction

The ambrosia beetle genus *Anisandrus* Ferrari, 1867, was erected for *Xyleborusdispar* (Fabricius, 1792) because of its antennal club and mouth parts which differ from other *Xyleborus* Eichhoff, 1864 species (Ferrari 1867). *Anisandrus* currently contains 35 species distributed through the Palearctic region, from Europe to Japan, and through the Oriental region to New Guinea and the Solomon Islands. A single species occurs in Madagascar, but the genus is not known from the African continent. Two species, *A.obesus* (LeConte, 1868) and *A.sayi* Hopkins, 1915, are indigenous to the Nearctic region, and two Palearctic species, *A.dispar* (Fabricius, 1792), and *A.maiche* (Kurentsov, 1941) have been introduced to and established in the USA (Wood 1977; Rabaglia et al. 2009; Gomez et al. 2018). *Anisandrusmaiche* is also established in Italy and Ukraine (Nikulina et al. 2015; Colombari et al. 2022). In Thailand, six *Anisandrus* species were previously recorded (Hutacharern and Tubtim 1995; Beaver and Liu 2010; Beaver et al. 2014; Smith et al. 2020), but one species, *A.apicalis* (Blandford, 1894) must be removed from the fauna following the recognition of closely similar species with which it was previously confused (see below). In the present study, we describe five new species and report one new species from Thailand, increasing the diversity of the Thai fauna to 11 *Anisandrus* species and that of the genus to 40. We also provide a key and synoptic list of the *Anisandrus* of Thailand.

## ﻿Materials and methods

Specimens were collected at 27 study sites in 24 conservation areas across all regions of Thailand as detailed and illustrated by Sittichaya and Smith (2022), with the addition of 10 study sites in the Tanaosi (Tenasserim) mountain range in western Thailand between September and December 2022 using the same collecting methods. Photographs were taken with a Canon 5D digital camera with a Canon MP-E 65 mm macro lens (Canon, Tokyo, Japan) and StackShot-Macrorail (Cognisys, Traverse City, Michigan, USA). The photos were then combined with Helicon Focus v. 6.8.0. (Helicon Soft, Kharkiv, Ukraine) and all photos were improved with Adobe Photoshop CS6 (Adobe Systems, San Jose, California, USA). The antennal and pronotum types and characters follow those proposed by Hulcr et al. (2007) and subsequently elaborated by Smith et al. (2020). Length was measured from pronotal apex to the apex of the declivity, and width was measured at the widest part of the specimen. Pronotal length included the anterior serrations and elytral length was measured from the anterior margin to the apex along the elytral medial suture. Pedicel is excluded from the number of funicle segments.

**Abbreviations used for entomological collections**:

**MSUC**Albert J. Cook Arthropod Research Collection, Michigan State University, East Lansing, USA

**NHMW**Naturhistorisches Museum Wien, Austria

**QSBG** Queen Sirikit Botanical Garden, Chiang Mai, Thailand

**RABC** Roger A. Beaver collection, Chiang Mai, Thailand

**THNHM** Natural History Museum of the National Science Museum, Thailand

**WSTC** Private collection of Wisut Sittichaya, Songkhla, Thailand

## ﻿Taxonomic treatment

### ﻿Xyleborini LeConte, 1876

#### ﻿ Anisandrus

Taxon classificationAnimaliaColeopteraCurculionidae

Ferrari, 1867

7DC14744-7D34-5E8A-9C9C-C85BA1F5B929

Anisandrus Ferrari, 1867: 24.

##### Type species.

*Apatedispar* Fabricius, 1792, by monotypy.

##### Differential diagnosis.

Antennal club obliquely truncate, type 1 (except *A.achaete* Smith, Beaver & Cognato, 2020, which is type 2) (Hulcr et al 2007; Smith et al. 2020), club taller than wide (except *A.achaete* wider than tall); procoxae contiguous or narrowly separated; protibiae slender, obliquely or distinctly triangular, outer margin with 5−8 large socketed denticles on distal half, posterior face flat, unarmed, or with a few small granules; mesonotal mycangial tufts present (except *A.achaete*, *A.carinensis* (Eggers, 1923), *A.paragogus* Smith, Beaver & Cognato, 2020, and *A.uniseriatus* sp. nov.) along the pronotal base either as a small tuft the length of the scutellum and directly opposite it or extending laterally from the scutellum to striae 3 and with elytral base broadly, shallowly emarginated from the scutellum to striae 3; pronotum anterior margin with a row of serrations, pronotum lateral margins obliquely costate (Smith et al. 2020).

Some *Anisandrus* species have a median pair of pronotal serrations larger than the remaining serrations and superficially resemble *Cnestus* Sampson species. The genera are easily separated by the lateral margin of the pronotum which is costate in *Anisandrus* and carinate in *Cnestus*.

### ﻿New species

#### ﻿ Anisandrus montanus

Taxon classificationAnimaliaColeopteraCurculionidae

Sittichaya, Smith & Beaver sp. nov.

5AFB2CC2-8C65-5364-B661-E11D001AEE5B

https://zoobank.org/A68B1284-FBA2-4172-AE79-02DBACC5BDD9

Fig. 1

##### Type materials.

***Holotype***: female, Thailand, Chiangmai Province, Chom Thong District, Doi Inthanon National Park, 18°32'03.1"N, 98°29'55.2"E, 1680m, high montane forest, ethanol-baited traps, 01.vi.[20]20, W. Sittichaya (MSUC). ***Paratypes***: same as holotype except: 18°35'10.5"N, 98°29'13.1"E, 2,550 m, 01.iv.[20]19, W. Sittichaya (2, WSTC; 1, THNHM); Chiang Mai, Doi Inthanon NP, Kaew Maepan Trail, 18°33.162'N, 98°28.810'E, 2250 m, Malaise trap, 10–17.xi.2006, Y. Areeluck (1, RABC); as previous except: 18°35.361'N, 98°29.157'E, summit forest, 2500 m, 9–16.viii.2006 (1, RABC); as previous except: 6–13.ix.2006 (1, QSBG).

**Figure 1.**
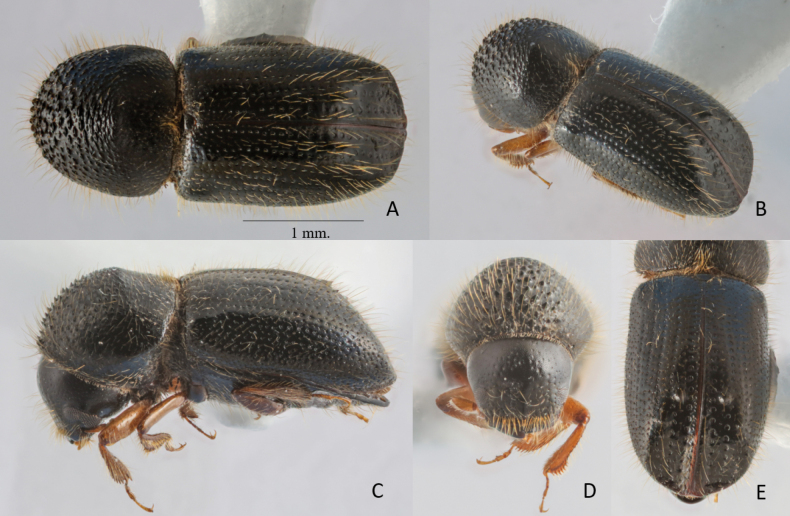
*Anisandrusmontanus* sp. nov. holotype female **A** dorsal view **B** postero-lateral view **C** lateral view **D** frons **E** declivital face.

##### Similar species.

*A.eggersi* (Beeson, 1930), *A.improbus* (Sampson, 1913).

##### Differential diagnosis.

2.80–2.92 mm long (mean 2.86, *n* = 4); 2.33–2.43× as long as wide. This species is similar to *A.eggersi*, but the body is smaller and more elongate, the declivity is less laterally broadened, and the posterolateral margin is not feebly costate. It is also similar to *A.improbus* but is distinguished by the following characteristics (*A.improbus* given first): sparse pubescence vs moderate pubescence, body 3.3–3.4 mm long vs 2.80–2.92 mm long, and body form more elongate, 2.43−2.54× as long as wide, vs stouter, 2.33–2.43× as long as wide.

##### Description.

**Female.** 2.80–2.92 mm long (mean 2.86, *n* = 4); 2.33–2.43× as long as wide. Body shiny and black, except appendages yellowish brown; body moderately densely covered with long, yellowish-brown, hair-like setae. ***Head***: epistoma entire, transverse, with a row of hair-like setae; setae moderately long, sparse. Frons feebly convex to upper level of eyes, smooth, moderately shining, rather sparsely punctured, except close to epistoma; punctures bearing long, fine, hair-like setae. Frons with a weakly elevated, glabrous median ridge from epistoma to mid-point; above the eyes slightly domed, less shiny, coriaceous. Eyes shallowly emarginate just above antennal insertion, upper part smaller than lower part. Submentum triangular, large, slightly impressed. Antennal scape regularly thick, slightly longer than club (1.1:1). Pedicel as wide as scape, half as long as funicle. Funicle 4-segmented, segment 1 shorter than pedicel. Club longer than wide, obliquely truncate, type 1; segment 1 corneous, encircling anterior face, with sharp marginal carina; segment 2 narrow, concave, corneous on anterior face; sutures absent on posterior face. ***Pronotum***: 0.90× as long as wide, in dorsal view rounded, type 1, sides convex, rounded anteriorly; anterior margin with a row of six small serrations; serrations not larger than asperities behind. In lateral view short and tall, type 3, disc as long as anterior slope, summit at midpoint; summit from lateral view weakly raised, disc flat. Anterior slope with moderately spaced, medium-sized, coarse asperities, becoming lower and more strongly transverse towards summit. Disc alutaceous, subshining, sparsely granulate-punctate, with a semi-recumbent, moderately long, fine, forwardly directed, hair-like seta arising from just anterior to each granule. Lateral margins obliquely costate; costa long, slightly elevated. Base slightly, broadly concave; posterior angles angulate. Mycangial tuft present along basal margin; tuft moderately setose, approximately equal to width of scutellum. ***Elytra***: 1.44× as long as wide, 1.63× as long as pronotum. Scutellum moderate in size, flat. Base transverse, edge oblique, humeral angles rounded, parallel-sided in basal 5/8, then broadly rounded to apex; surface shiny. Disc shiny, moderately convex, without transverse saddle-like depression; striae with broad, shallow punctures separated by 1/2 diameter of a puncture, setose, setae slightly longer than two diameters of a puncture, semi-recumbent, hair-like; interstriae flat, 2–3× as wide as striae, punctate; punctures uniseriate, minute, setose; setae long, erect, hair-like, becoming longer posteriorly; interstriae 2 weakly raised near declivital summit, so that first striae and interstriae appear shallowly sulcate. Declivity occupying approximately 1/3 of elytra, evenly rounded, declivital face narrow, opalescent, weakly bisulcate, moderately impressed between interstriae 1 and 3 in upper part, interstriae 3 weakly inflated near summit, flat below. Declivital striae weakly impressed, strial punctures moderately larger and deeper than those of disc, with setae as described for disc; interstriae impunctate, sparsely minutely granulate; setae 2–3× width of interstriae 2, erect, hair-like; interstriae 2 either as wide as or narrower than interstriae 3 at midpoint of declivity. Declivital summit armed with a small, sharp, backwardly pointed spine on interstriae 2 and 3; spine on interstriae 2 stronger. Posterolateral margin costate to interstriae 5. ***Legs***: procoxae contiguous. Protibiae obliquely triangular, broadest at apical 1/3; posterior face of protibiae punctate, with some punctures near base and inner margin with small, sparse granules; apical ½ of outer margin with six large, socketed denticles, their length longer than basal width. Meso- and metatibiae flattened; outer margins evenly rounded with nine and 10 large socketed denticles, respectively.

**Male.** Unknown.

##### Etymology.

Latin adjective *montanus*, found on mountains. The species is known only from Doi Inthanon, the highest mountain in Thailand, at 1680‒2550 m.

##### Distribution.

Thailand (Chiangmai Province).

##### Biology.

This species prefers montane forest.

##### Remarks.

Three of the paratypes listed above were previously reported as *A.apicalis* by Beaver et al. (2014).

#### ﻿ Anisandrus phithakpa

Taxon classificationAnimaliaColeopteraCurculionidae

Sittichaya, Smith & Beaver sp. nov.

75F0F0C6-926E-5598-82C0-C4266FF80557

https://zoobank.org/80EC9C36-187D-40F2-A37C-A62B879D2C47

Fig. 2

##### Type materials.

***Holotype***: female, Thailand, Phetchaburi Province, Kaeng Krachan District, Kaeng Krachan National Park, 12°49'43.6"N, 99°21'45.2"E, 900 m, low montane forest, ex *Lithocarpus* sp., 04.x.22, W. Sittichaya (MSUC). ***Paratypes***: Kanchanaburi Province, Thong Pha Phum District, Thong Pha Phum National Park, 14°41'40.6"N, 98°23'51.9"E, 940 m, low montane forest, ethanol-baited trap, 11.xii.22, W. Sittichaya (1, WSTC; 1, THNHM; 1, RABC).

**Figure 2.**
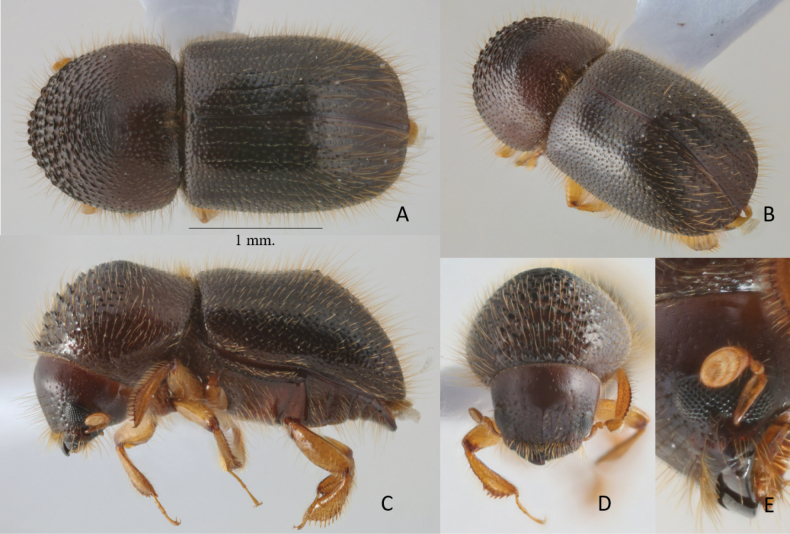
*Anisandrusphithakpa* sp. nov. holotype female **A** dorsal view **B** postero-lateral view **C** lateral view **D** frons **E** antenna.

##### Similar species.

*A.apicalis*, *A.congruens* Smith, Beaver & Cognato, 2020, *A.cristatus* (Hagedorn, 1908).

##### Differential diagnosis.

2.88–3.02 mm long (mean 2.96 mm, *n* = 4); 2.06–2.17× as long as wide. Stout species; elytral disc saddle-like, bearing a pair of small spines on interstriae 2. Declivity broad; declivital face with striae 1 and 2 weakly impressed; interstriae 1 and 3 slightly elevated; posterolateral margin rounded, costate only near apex. The species is similar to *A.apicalis*, *A.congruens*, and *A.cristatus*. *Anisandruscongruens* and *A.cristatus* are distinguished by the presence on declivital interstriae 3 of a row of 5‒7 regularly spaced, backwardly directed, sharply pointed spines; in *A.phithakpa* and *A.apicalis* these are reduced to small granules. *Anisandrusphithakpa* is distinguished from *A.apicalis* by the following characters (*A.phithakpa* given first): declivital interstriae with a pair of minute, pointed granules on interstriae 1, and an equally sized pair on interstriae 2 and 3 vs no granules on interstriae 1, and interstriae 2 with a pair of small, backwardly directed, sharply pointed teeth which are considerably larger than the pointed granules on interstriae 3.

##### Description.

**Female.** 2.88–3.02 mm long (mean 2.96 mm; *n* = 4); 2.06–2.17× as long as wide (mean 2.12× as long as wide; *n* = 4). ***Body colors***: dark brown to black, appendages paler brown. ***Head***: epistoma entire, transverse, with a row of hair-like, moderately long, sparse setae. Frons weakly convex to upper level of eyes, reticulate, subshining, with sparse widely separated, small, shallow, setose punctures each with a long, erect hair-like seta. Medial area inconspicuous, feebly convex (flat in one paratype), glabrous. Eyes shallowly emarginate just above antennal insertion, upper part smaller than lower part. Submentum moderate, distinctly triangular, slightly impressed. Antennal scape regularly thick, 1.2× as long as club. Pedicel as wide as scape, shorter than funicle. Funicle 4-segmented; segment 1 shorter than pedicel. Club longer than wide, obliquely truncate, type 1; segment 1 corneous, encircling anterior face; segment 2 narrow, concave, corneous; sutures absent on posterior face. ***Pronotum***: 0.98× as long as wide, in dorsal view rounded, type 1; sides convex, rounded anteriorly; anterior margin with a row of eight small serrations of same size as asperities above; in lateral view, short and tall, type 3; disc as long as anterior slope, summit at midpoint. Anterior slope with densely spaced, very large, coarse asperities, becoming lower and more strongly transverse towards summit. Disc alutaceous, subshiny with moderately dense, large, shallow punctures; punctures with moderate, semi-recumbent, hair-like setae; some longer hair-like setae at margins. Lateral margins obliquely costate. Base transverse with posterior angles rounded. Mycangial tuft present along basal margin; tuft moderately setose, approximately the width of scutellum. ***Elytra***: 1.5× as long as wide, 1.5× as long as pronotum. Scutellum broad, large, linguiform, flush with elytra, flat, shiny. Elytral base transverse; edge oblique; humeral angles rounded; elytra parallel-sided in basal ½, then broadly rounded to apex; surface shiny. Disc shiny, with a distinct medial, transverse, saddle-like depression; depressed areas opalescent; striae not impressed; with broad shallow punctures separated by areas less than a diameter of a puncture, setose; setae 2–3× as long as a puncture, recumbent, hair-like; interstriae flat, punctate, with 2 or 3 confused lines of minute punctures, setose; setae long, 1–1.5× width of interstriae 2, erect, hair-like, unarmed by granules. Declivity occupying approximately 1/3 elytra; apex evenly rounded; declivital summit with a pair of minute, pointed granules on interstriae 1, and a slightly larger pair on both interstriae 2 and 3 placed progressively further towards apex; declivital face feebly bisulcate; striae 1 and 2 impressed; interstriae 3 inflated and armed, with 2 or 3 minute granules; strial punctures of similar size and depth to those of disc, bearing setae as described for disc; interstriae impunctate, sparsely, minutely granulate; setae 2× width of interstriae 2, erect, hair-like; interstriae 2 as wide as or narrower than interstriae 3 at midpoint of declivity. Posterolateral margin costate to interstriae 5. ***Legs***: procoxae contiguous; prosternal coxal piece short, inconspicuous. Protibiae obliquely triangular, broadest at apical 1/3; posterior face inflated, punctate, punctures minute; apical 1/2 of outer margin with six socketed denticles, their length 2× their basal as basal width. Meso- and metatibiae flattened; outer margins evenly rounded with seven and eight long slender socketed denticles, respectively.

**Male.** Unknown.

##### Etymology.

Thai (พิทักษ์ป่า) “Phithakpa”, forest rangers. The species name indicates our deep appreciation for Thai forest rangers for their hard and selfless work to protect conservation areas in Thailand. Noun in apposition.

##### Distribution.

Thailand (Kanchanaburi and Phetchaburi provinces).

##### Biology.

Recorded from *Lithocarpus* sp. (Fagaceae).

##### Remarks.

The paler body colors (brown) of the holotype and some paratypes suggest that they are teneral. One paratype has a consistently dark-brown body with pale appendages.

#### ﻿ Anisandrus tanaosi

Taxon classificationAnimaliaColeopteraCurculionidae

Sittichaya, Smith & Beaver sp. nov.

FAE32135-40D0-50CB-ADB3-6C38ED257E25

https://zoobank.org/4B554589-BDE9-460C-AEAA-6D7208213A80

Fig. 3

##### Type materials.

***Holotype***: female, Thailand, Phetchaburi Province, Kaeng Krachan District, Kaeng Krachan National Park, 12°49'43.6"N, 99°21'45.2"E, 900 m, low montane forest, ex. *Lithocarpus* sp., 04.x.22, W. Sittichaya (MSUC). ***Paratype***: Kanchanaburi Province, Thong Pha Phum District, Thong Pha Phum National Park, 14°41'40.6"N, 98°23'51.9"E, 940 m, low montane forest, ethanol-baited trap, 11.xii.22, W. Sittichaya (1 WSTC).

**Figure 3.**
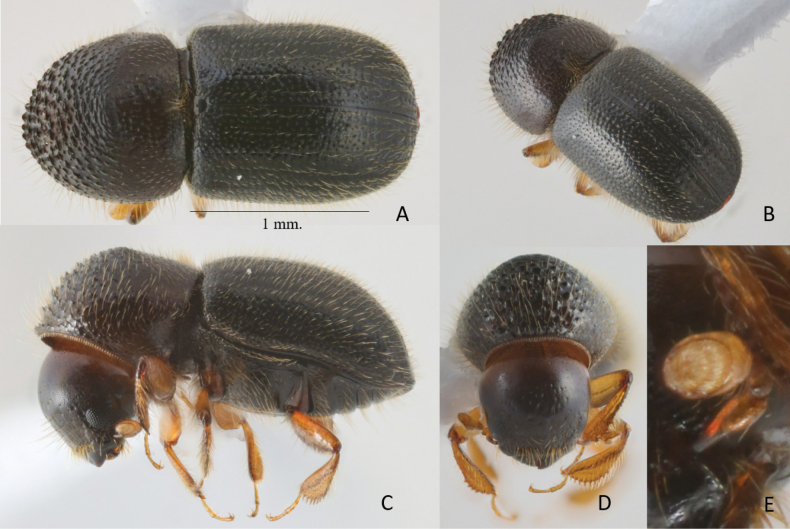
*Anisandrustanaosi* sp. nov. holotype female **A** dorsal view **B** postero-lateral view **C** lateral view **D** frons **E** antenna.

##### Similar species.

*A.auco* Smith, Beaver & Cognato, 2020, *A.cryphaloides* Smith, Beaver & Cognato, 2020, *A.triton* sp. nov.

##### Differential diagnosis.

2.31–2.70 mm long (mean 2.5; *n* = 2); 2.20–2.25× as long as wide (mean = 2.23; *n* = 2). Small and stout species. Pronotal anterior margin slightly angularly projecting; elytral disc convex, without a saddle-like impression, declivital summit armed with a pair of minute, spinulose granules on interstriae 2, declivital face feebly convex, unarmed. Posterolateral margin rounded, costate only near apex. The species is similar to *A.auco* and *A.cryphaloides*. It can be distinguished from *A.auco* by the following characters (*A.tanaosi* given first): smaller size (2.3‒2.7 mm long vs 2.9‒3.0 mm); anterior margin of pronotum with seven moderately sized asperities vs four large coarse asperities; more steeply sloping elytral declivity occupying 3/8 of elytral length vs more gently sloping occupying 3/5 of length; dark-brown to black body vs light brown to reddish brown. It can be distinguished from *A.cryphaloides* by the following characters (*A.tanaosi* given first): pronotum in dorsal view type 0, anterior margin only slightly projecting, with asperities of equal size vs pronotum more strongly conical, type 6, median pair of asperities larger than lateral pairs.

##### Description.

**Female.** 2.31–2.70 mm long (mean 2.5; *n* = 2); 2.20–2.25× as long as wide (mean 2.23; *n* = 2). Body dark brown to black, head and prothorax dark brown, elytra and venter black, appendages yellowish brown. Antennae and legs light brown. Body densely covered with greyish-brown setae. ***Head***: epistoma entire, transverse, with a row of short hair-like setae, setae sparse. Frons feebly convex to upper level of eyes, weakly reticulate, rugulose-punctate, some rugulosities forming longitudinal lines; each puncture with a moderately long, fine, hair-like setae; a weak, impunctate median ridge extends to upper level of eyes. Eyes feebly emarginate just above antennal insertion, upper part slightly smaller than lower part. Submentum triangular, small, slightly impressed. Antennal scape regularly thick, short, as long as club. Pedicel as wide as scape, shorter than funicle. Funicle 4-segmented, segment 1 as long as pedicel. Club longer than wide, obliquely truncate, type 1; segment 1 corneous, encircling anterior face; segment 2 concave, soft and narrow; sutures absent on posterior face. ***Pronotum***: 0.90× as long as wide. In dorsal view, type 0, feebly conical anteriorly, sides convex; anterior margin with a row of seven small, slightly protruding serrations, equal in size to those on anterior slope. In lateral view type 3, short and tall; disc as long as anterior slope, summit at midpoint. Anterior slope with moderately densely spaced, large coarse asperities, becoming lower and more strongly transverse towards summit. Disc alutaceous, subshining with moderately dense, minute granulate punctures, each bearing a short, semi-recumbent, hair-like seta, some longer hair-like setae at margins. Lateral margins obliquely costate. Base transverse, posterior angles angularly rounded. Mycangial tuft present along basal margin, tuft moderately setose, approximately the width of scutellum. ***Elytra***: 1.22× as long as wide, 1.42× as long as pronotum. Scutellum broad, large, linguiform, flush with elytra, flat, shiny. Elytral base transverse, edge oblique, humeral angles rounded, parallel-sided in basal 2/3, then broadly rounded to apex. Disc subshiny, broadly convex; striae not impressed, with small, shallow, setose punctures separated by 1.5–2× diameters of a puncture, setae 3× as long as diameter of punctures, recumbent, hair-like; interstriae flat; punctures strongly confused, without granules, setose; setae short, as long as strial setae, erect hair-like. Declivity occupying approximately 3/8 elytra; summit with a pair of spinulose granules on interstriae 2; declivital face feebly convex above, flattened below from interstriae 1–3; striae weakly impressed; strial punctures somewhat larger and deeper than those of disc; interstriae sparsely uniseriate punctate, setae 2× width of an interstria, erect, hair-like. Posterolateral margin rounded, unarmed by granules, costate only close to apex. ***Legs***: procoxae contiguous; prosternal coxal piece short, inconspicuous. Protibiae obliquely triangular, broadest at apical 1/3; posterior face minutely granulate; apical 1/3 of outer margin with six small, socketed denticles, their length as long as basal width. Meso- and metatibiae flattened; outer margins evenly rounded with eight large, socketed denticles.

**Male.** Unknown.

##### Etymology.

Tanaosi (ตะนาวศรี), Thai name of the Tenasserim mountain range, in reference to the collection locality of the holotype. Noun in apposition.

##### Distribution.

Thailand (Kanchanaburi and Phetchaburi provinces).

##### Biology.

Unknown.

#### ﻿ Anisandrus triton

Taxon classificationAnimaliaColeopteraCurculionidae

Sittichaya, Smith & Beaver sp. nov.

8618E576-5C70-5125-AAFC-FB4F65F9E727

https://zoobank.org/411AD77C-E814-4329-9ACC-145754A53EDB

Fig. 4

##### Type material.

***Holotype***: female, Thailand, Kanchanaburi Province, Thong Pha Phum District, Thong Pha Phum National Park, 14°41'40.6"N, 98°23'51.9"E, 940m, low montane forest, ethanol-baited trap, 11.xii.22, W. Sittichaya (MSUC).

**Figure 4.**
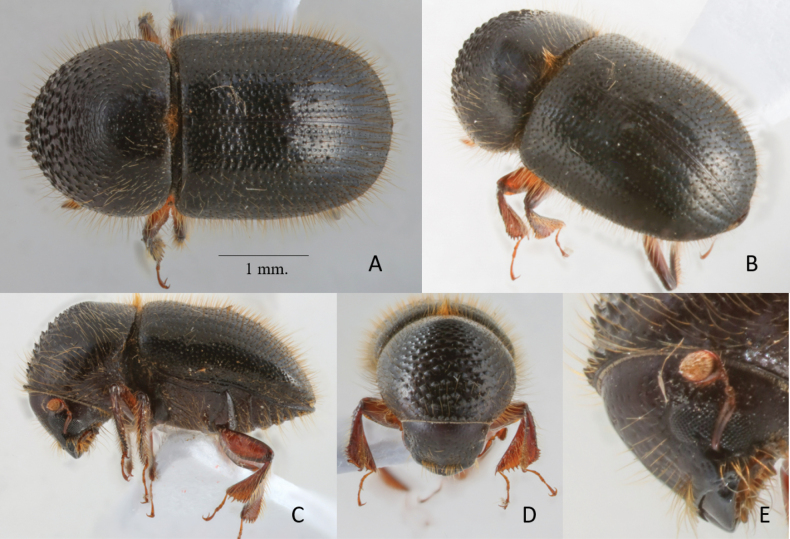
*Anisandrustriton* sp. nov. holotype female **A** dorsal view **B** postero-lateral view **C** lateral view **D** frons **E** antenna.

##### Similar species.

*Anisandrusauco*, *A.cryphaloides*.

##### Differential diagnosis.

4.22 mm long; 1.94× as long as wide. Large, broad, stout species. Elytral disc with a saddle-like, weak impression on middle of disc; declivity longer than disc; interstriae 2 armed with two pairs of spines, backwardly incurved on declivital summit and one additional smaller sized spine on upper portion of declivital face; interstriae 3 armed with a row of 3–5 unequally sized spines and granules; declivital face feebly convex, apex broadly rounded; posterolateral margin rounded, with a short costa near apex. The species is similar to *Anisandrusauco*, *A.cryphaloides*, and *A.tanaosi*. It can be distinguished from them by the following characters (*A.triton* given first): greater size (4.2 mm long vs 2.1‒3.0 mm) and stouter body (1.94× longer than wide vs 2.2‒2.4×; elytral disc with a weak, saddle-like depression vs elytral disc flat; upper margin of the elytral declivity with a pair of backwardly directed, sharply pointed spines vs a pair of minute, pointed granules.

##### Description.

**Female.** 4.22 mm long (*n* = 1); 1.94× as long as wide. Body black except appendages brown; body densely covered with long, erect, yellowish-brown, hair-like setae. ***Head***: epistoma entire, transverse, with a row of short and sparse, hair-like setae, sparser in the middle and on lateral margins below eyes. Frons with a weak median ridge extending to upper margin of eyes, weakly impressed on each side near epistoma, becoming flattened and weakly convex above, reticulate, subshining, with sparse, large, shallow, punctures, each puncture bearing a shorter, finer, erect hair-like seta than those on epistoma; punctures becoming smaller and shallower towards vertex. Eyes large, feebly emarginate just above antennal insertion; upper part of eyes much smaller than lower part. Submentum transversely long, narrowly triangular, slightly impressed. Antennal scape slender, 1.4× as long as club. Pedicel as wide as scape, shorter than funicle. Funicle 4-segmented, segment 1 as long as pedicel. Club longer than wide, obliquely truncate, type 1; segment 1 corneous, encircling anterior face; segment 2 narrow, corneous on anterior face only; sutures absent on posterior face. ***Pronotum***: 0.83× as long as wide. In dorsal view, between type 0 and type 6, sides convex, strongly narrowed anteriorly; anterior margin with a row of seven medium-sized serrations. In lateral view, short and tall, type 3; disc slightly shorter than anterior slope. Anterior slope with moderately dense, large, coarse asperities, becoming lower and more strongly transverse towards summit. Disc convex, moderately shiny with moderately dense, minute, punctures bearing two types of setae: moderately long, erect, hair-like setae and short, semi-recumbent, hair-like setae; some longer, hair-like setae at margins. Base transverse; posterior angles broadly rounded. Mycangial tuft present along basal margin; tuft dense, long, setose, approximately 2× width of scutellum. ***Elytra***: 1.18× as long as wide, 1.63× as long as pronotum. Scutellum small, broad, linguiform, shiny, slightly convex, flush with elytra. Elytral base transverse, edge oblique, humeral angles rounded, parallel-sided in basal ½, then broadly rounded to apex; surface shining. Disc shallowly, transversely impressed; striae not impressed, with medium-sized, shallow punctures separated by the diameter of a puncture; strial setae 1.5× as long as punctures, semi-recumbent, hair-like; discal interstriae 1 and 3 flat, interstriae 4 and 5 weakly convex; near upper margin of declivity, interstriae 1–5 weakly convex; interstriae biseriate punctate, punctures minute, shallow, each bearing an erect hair-like seta; setae on disc as long as interstrial width, some longer setae present on lateral and apical margins of elytra; punctures on lateral margins and near declivital summit replaced by small granules. Declivity occupying approximately 1/2 elytra, evenly rounded, declivital face convex; striae feebly impressed, strial punctures the same size and depth as those of disc; interstriae feebly convex, 1–3 of equal width, biseriate granulate; setae 2× width of an interstria, erect, hair-like; declivital interstria 2 armed with two pairs of spines; the larger on declivital summit backwardly hooked, the smaller on upper portion of declivital face pointed; interstria 3 armed by a row of 5 spinulose granules, the upper two pairs slightly backwardly hooked. Posterolateral margin of declivity rounded, with a short costa near apex, unarmed by granules. ***Legs***: procoxae slightly separated; prosternal coxal piece short, inconspicuous. Protibiae obliquely triangular, broadest at apical 1/3; posterior face inflated, unarmed; apical 1/2 of outer margin with six moderately sized socketed denticles, length approximately equal to basal width. Meso- and metatibiae flattened; outer margins evenly rounded each with eight moderately sized socketed denticles.

**Male.** Unknown.

##### Etymology.

Ancient Greek, *triton* is a fish-tailed sea-god, named after a veteran vehicle used in beetle surveys by the senior author.

##### Distribution.

Thailand (Kanchanaburi Province).

##### Biology.

Unknown.

#### ﻿ Anisandrus uniseriatus

Taxon classificationAnimaliaColeopteraCurculionidae

Sittichaya, Smith & Beaver sp. nov.

316E1949-8C87-56D6-BDF0-67E93C658D83

https://zoobank.org/A00B2A08-2C6F-4922-8620-4EBDF881F1A4

Fig. 5

##### Type material.

***Holotype***, female, Thailand, Nan Province, Pua District, Doi Phu Kha National Park, 19°10'27.4"N, 101°06'19.7"E, 1660 m, montane forest, ethanol-baited traps, 01.viii.19 W. Sittichaya (MSUC). ***Paratypes***: same as holotype except: 30.vi.19 (2) (1, NHMW; 1, RABC); same as holotype except: 10.x.19 (2) (1, THNHM; 1, WSTC).

**Figure 5.**
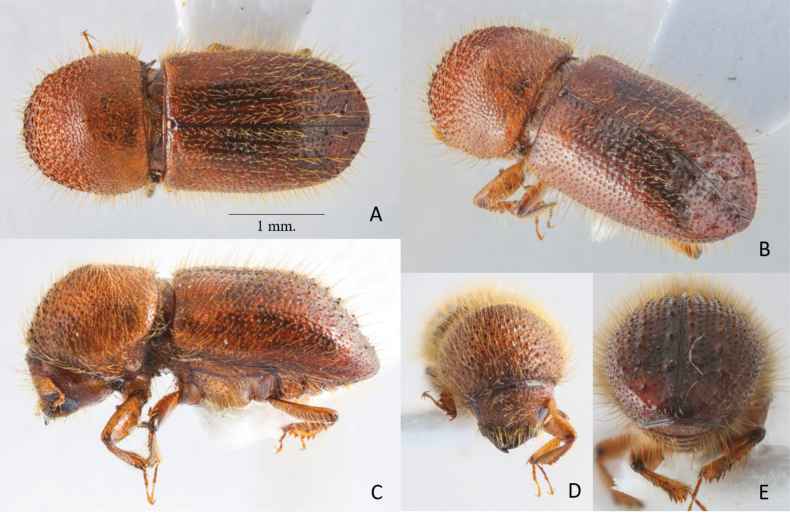
*Anisandrusuniseriatus***sp. nov.** holotype female **A** dorsal view **B** lateral view **C** postero-lateral view **D** frons **E** venter and antennae **F** declivital face.

##### Similar species.

*Anisandruscarinensis*.

##### Differential diagnosis.

4.0–4.32 mm long (mean 4.20 mm; *n* = 5); 2.32–2.60× as long as wide (mean 2.44 mm; *n* = 5). Species large, robust; body yellowish brown, covered with long erect hair-like setae; pronotal anterior margin broadly rounded and armed with a row of serrations; mesonotal mycangial tuft absent; antennal club type 1 with segment 1 encircling anterior face; elytral disc with a weak transverse saddle-like depression; interstriae uniseriate granulate; declivital interstriae 1 and 3 armed by four or five unequally sized tubercles; elytral apex angularly rounded.

This species is closely related to *A.carinensis* but is distinguished by the following characteristics (*A.uniseriatus* given first): discal interstria uniseriate granulate-punctate vs biseriate granulate-punctate; angularly rounded apex vs broadly rounded elytral apex.

##### Description.

**Female.** 4.0–4.32 mm long (mean 4.20 mm; *n* = 5); 2.32–2.60× as long as wide (mean 2.44 mm; *n* = 5). Body yellowish brown to reddish brown, declivity darker. Head, legs, and antennae light brown. ***Head***: epistoma entire, transverse, with a row of golden, long and thick hair-like setae. Frons flat to upper level of eyes, shining, with sparse small granules, each with a fine, long, erect hair-like seta; median line narrowly elevated to upper margin of eyes, glabrous strongly shining. Vertex convex, reticulate, with longitudinal shallow rugae and punctures. Eyes shallowly emarginate just above antennal insertion, upper part smaller than lower part. Submentum large, distinctly triangular, slightly impressed. Antennal scape regularly thick, as long as club. Pedicel as wide as scape, shorter than funicle. Funicle 4-segmented, segment 1 shorter than pedicel. Club wider than long, obliquely truncate, type 1; segment 1 corneous, transverse, occupying basal 2/5, encircling anterior face; segment 2 narrow, concave, corneous; sutures absent on posterior face. ***Pronotum***: 0.89× as long as wide. In dorsal view basic, type 2, sides parallel in basal 1/2, rounded anteriorly; anterior margin without distinct serrations. In lateral view basic, type 0, disc as long as anterior slope, summit at apical 2/5. Anterior slope with densely placed asperities of very variable size, becoming lower and more strongly transverse towards summit. Disc shining, with densely placed, minute asperities and granules, arranged approximately concentrically behind summit; vestiture of long, erect hair-like setae interspersed with shorter, more abundant, semi-recumbent setae directed antero-medially; some longer hair-like setae on convex lateral margins. Base transverse, posterior angles rounded. Mycangial tuft absent. ***Elytra***: 1.55× as long as wide, 1.75× as long as pronotum. Scutellum narrow, moderately sized, linguiform, flush with elytra, flat, shiny. Elytral bases transverse, edge oblique, humeral angles rounded; elytra parallel-sided in basal 2/3, then broadly rounded to apex; surface shiny. Disc with a very slight, transverse, saddle-like depression in middle; only striae 1 impressed, its punctures coarse, shallow, regularly placed, separated by about the diameter of a puncture, and bearing fine, moderately long, hair-like setae; interstriae finely punctate, with some punctures (approximately every second puncture) granulate; interstria 1 uniseriately punctured, but with 2‒3 rows of punctures where widened posteriorly close to declivity; interstria 2 uniseriately punctate along its length; interstriae 3‒5 biseriately punctate close to base, uniseriate posteriorly; granulate punctures on interstriae bearing long, fine, erect hairlike setae, non-granulate punctures with shorter, semi-recumbent setae. Declivity occupying approximately 1/3 of elytra, steeply sloping; declivital face weakly bisulcate between raised interstriae 1 and 3; strial punctures larger and deeper than those of disc; interstriae uniseriate with some punctures replaced by granules or tubercles; interstriae 1 widest at mid-declivity, with 4‒6 larger pointed tubercles, and some smaller granules; interstriae 2 with a few small granules at top of declivity only, narrowed towards apex; interstriae 3 with 5‒7 pointed tubercles, a little smaller than those on interstriae 1; interstrial tubercles and granules bearing long, erect setae 1.5× width of interstriae 2; interstrial punctures with finer, shorter setae. Posterolateral margin of elytra rounded, costate only near apex, unarmed by granules. ***Legs***: procoxae contiguous, prosternal coxal piece tall and pointed. Protibiae distinctly triangular, broadest at apical 4/5, posterior face smooth; apical 1/2 of outer margin with five moderately sized socketed denticles, their length slightly longer than basal width. Meso- and metatibiae flattened, obliquely triangular, their apical 1/2 with 5- or 6-socketed denticles on outer margin.

**Male.** Unknown.

##### Etymology.

Latin *uniseriatus*: uni- meaning one; series meaning row, in reference to a single row of interstrial setae.

##### Distribution.

Thailand (Nan Province).

##### Biology.

Unknown.

### ﻿New country record

#### ﻿ Anisandrus carinensis

Taxon classificationAnimaliaColeopteraCurculionidae

(Eggers, 1923)

A3524A02-86C9-556C-91D5-E7D67386108B

Fig. 6

##### Differential diagnosis.

3.70–4.26 mm long (mean 4.08; *n* = 7); 2.27–2.35× as long as wide (mean 2.31×; *n* = 7). Moderate to large in size, robust form; body yellowish brown, covered with long erect hair-like setae; pronotal anterior margin broadly rounded and armed with a row of serrations on anterior margin of pronotum; mesonotal mycangial tuft absent; antennal club type 1 with segment 1 encircling anterior face; elytral disc with a weak transverse saddle-like depression; interstriae biseriate granulate; declivital interstriae 1 and 3 armed by four or five unequally sized tubercles; elytral apex broadly rounded.

**Figure 6.**
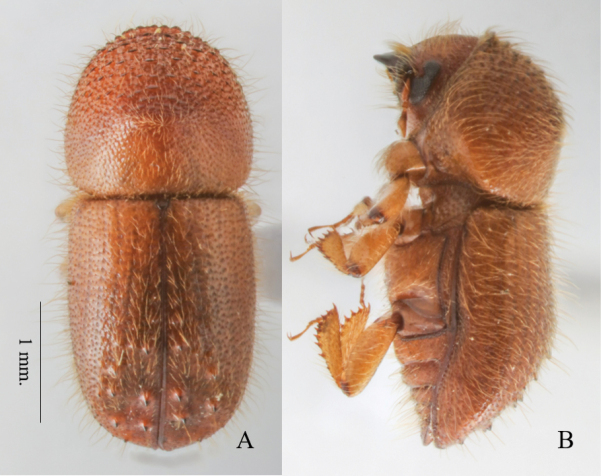
*Anisandruscarinensis* female. **A** dorsal view **B** lateral view.

##### Thai distribution.

S: Ranong Province, Suk Samran District, Klong Naka Wildlife Sanctuary, 9°27'42.8"N, 98°32'23.3"E, 320 m, tropical lowland forest, ethanol-baited traps, 01.iv.14 (4); Suratthani Province, Ban Ta Khun District, Khao Sok National Park, 8°55'25.6"N, 98°31'19.2"E, 380 m, tropical rain forest, ethanol-baited trap, 01.v.14 (10). W: Kanchanaburi Province, Thong Pha Phum District, Thong Pha Phum National Park, 14°41'40.6"N, 98°23'51.9"E, 940 m, montane forest, ethanol baited trap, 10.ix.22 (3); all W. Sittichaya.

##### Other distribution.

Myanmar (Smith et al. 2020).

### ﻿Correction to faunal list for Thailand

#### ﻿ Anisandrus apicalis

Taxon classificationAnimaliaColeopteraCurculionidae

(Blandford, 1894)

4D2C0013-6672-52F5-9D4F-EBCB515193F6

##### Notes.

Seven specimens were recorded from Chiang Mai province under this name by Beaver et al. (2014). Three of these specimens are now included in the closely similar species, *A.montanus* sp. nov. (see above). The other four specimens belong to *A.cristatus*, which was considered a synonym of *A.apicalis* at the time of Beaver et al.’s publication. It was reinstated as a distinct species by Smith et al. (2020). We know of no specimens of *A.apicalis* from Thailand, and the species must be removed from the faunal list.

## ﻿Discussion

*Anisandrus* has recently been the focus of intense study, with 18 species described since 2020, including those described here (Smith et al. 2020, 2022). The genus is diverse in montane forest habitats in Southeast Asia, and Thailand in particular (Table 1). There is no doubt that additional species await discovery in unsampled mountain ranges throughout Southeast Asia.

**Table 1.** Synoptic list and habitat types of the *Anisandrus* fauna of Thailand. References are to records of the species in Thailand. Thai distribution follows that of Sittichaya and Smith (2022) which lists the following abbreviations: C = Central; N = North; NE = Northeast; S = South.

Species	Thai distribution	Habitat types	References
*Anisandruscarinensis* (Eggers, 1923)	W: Kanchanaburi; S: Ranong, Suratthani	Tropical rain forest, low montane forest	This publication
*Anisandruscongruens* Smith, Beaver & Cognato, 2020	N: Chiang Mai, Nan	Montane forest	Smith et al. 2020; W. Sittichaya (unpublished)
*Anisandruscristatus* (Hagedorn, 1908)	N: Chiang Mai, Nan	Montane forest	Smith et al. 2020; W. Sittichaya (unpublished)
*Anisandruseggersi* (Beeson, 1930)	N: Chiang Mai, Nan; NE: Loei	Montane forest	Smith et al. 2020; W. Sittichaya (unpublished)
*Anisandrushirtus* (Hagedorn, 1904)	N: Chiang Mai, Nan; NE: Loei; S: Nakhon Sri Thammarat, Narathiwat, Phang Nga, Ranong, Songkhla, Suratthani, Trang	Dry dipterocarp forest, Montane forest, Tropical rain forest	Beaver and Liu 2010; Beaver et al. 2014
*Anisandrusmontanus* sp. nov.	N: Chiang Mai	Montane forest	This publication
*Anisandrusphithakpa* sp. nov.	C: Phetchaburi; W: Kanchanaburi	Low montane forest	This publication
*Anisandrustanaosi* sp. nov.	C: Phetchaburi; W: Kanchanaburi	Low montane forest	This publication
*Anisandrustriton* sp. nov.	W: Kanchanaburi	Low montane forest	This publication
*Anisandrusuniseriatus* sp. nov.	N: Nan	Montane forest	This publication
*Anisandrusursulus* (Eggers, 1923)	C: Chanthaburi, Nakhon Nayok, Phetchaburi; N: Chiang Mai, Tak; NE: Loei, Nakhon Ratchasima; S: Nakhon Sri Thammarat, Surat Thani	Dry dipterocarp forest, Dry evergreen forest, Montane forest, Tropical rain forest	Hutacharern and Tubtim 1995; Beaver et al. 2014

### ﻿Key to *Anisandrus* species present in Thailand (females only)

**Table:** 

1	Mycangial tuft present, just anterior and roughly equal in width to scutellum, lightly to moderately setose	**2**
–	Mycangial tuft absent	**10**
2	Interstriae 2 without spines or granules on upper margin of elytral declivity. Large. densely hairy species, 3.4–4.9 mm long. Median pair of asperities on anterior margin of pronotum distinctly larger than outer pair	**3**
–	Interstriae 2 with spines, spinulose granules or blunt tubercles on upper margin of elytral declivity. Usually smaller, less densely hairy species. Median pair of asperities on anterior margin of pronotum not distinctly larger than outer pair(s)	**4**
3	Larger, stouter species, 4.3–4.9 mm long, 1.9–2.0× longer than wide. Declivital striae not impressed	***A.ursulus* (Eggers)**
–	Smaller, more elongate species, 3.4–4.5 mm long, 2.1–2.5× longer than wide. Declivital striae impressed	***A.hirtus* (Hagedorn)**
4	Interstriae 2 with a sharp, hooked spine on summit of elytral declivity	**5**
–	Interstriae 2 with rounded or spinulose granules, never with a sharp, hooked spine on summit of elytral declivity	**8**
5	Large, stout species, 4.2 mm long, 1.95× as long as wide; elytral declivity weakly convex	***A.triton* sp. nov.**
–	Smaller, more elongate species, 2.6–3.7 mm long, 2.2–2.5× as long as wide; elytral declivity impressed, often bisulcate	**6**
6	Interstriae 3 on elytral declivity armed with a row of 4 or 5 backwardly pointed spines, its upper portion elevated to middle of declivity	**7**
–	Interstriae 3 on elytral declivity armed only with 1 or 2 backwardly pointed spines; upper portion of declivital interstriae flat, not elevated	***A.montanus* sp. nov.**
7	Smaller species, 2.6–2.8 mm long. Spines on declivital interstriae 3 sharply pointed but not backwardly hooked	***A.congruens* Smith, Beaver & Cognato**
–	Larger species, 3.3–3.7 mm long. Spines on declivital interstriae 3 sharply pointed and backwardly hooked	***A.cristatus* (Hagedorn)**
8	Declivital interstriae impunctate, punctures replaced by minute granules, especially on interstriae 1–3; declivital summit armed with two pairs of distinct spinulose granules on interstriae 2 and 3; posterolateral margin of elytra strongly or weakly costate to interstriae 5	**9**
–	Declivital striae punctate; declivital summit armed only by a pair of minute, spinulose granules on interstriae 2; posterolateral margin of elytra costate only close to apex	***A.tanaosi* sp. nov.**
9	Elytral disc with a weak, transverse, saddle-like depression; posterolateral margin of elytra weakly costate	***A.phithakpa* sp. nov.**
–	Elytral disc without a weak, transverse, saddle-like depression; posterolateral margin of elytra strongly costate	***A.eggersi* (Beeson)**
10	Interstriae on elytral declivity irregularly biseriate granulate-punctate, elytral apex broadly rounded	***A.carinensis* (Eggers)**
–	Interstriae on elytral declivity uniseriate granulate-punctate, elytral apex angulately rounded	***A.uniseriatus* sp. nov.**

## Supplementary Material

XML Treatment for Anisandrus

XML Treatment for Anisandrus montanus

XML Treatment for Anisandrus phithakpa

XML Treatment for Anisandrus tanaosi

XML Treatment for Anisandrus triton

XML Treatment for Anisandrus uniseriatus

XML Treatment for Anisandrus carinensis

XML Treatment for Anisandrus apicalis

## ﻿Additional information

### Author ORCIDs

Wisut Sittichaya https://orcid.org/0000-0001-6200-1285

Sarah M. Smith https://orcid.org/0000-0002-5173-3736

Roger A. Beaver https://orcid.org/0000-0003-1932-3208

### Data availability

All of the data that support the findings of this study are available in the main text.
